# Non-polar components in PM_2.5_ increase matrix remodeling of CRS by up-regulating CEMIP in nasal fibroblasts

**DOI:** 10.3389/fgene.2025.1672729

**Published:** 2025-10-23

**Authors:** Jiayao Zhou, Ying Zhu, Huilin Hu, Ru Tang, Shiyao Zhang, Yuelong Gu, Song Mao, Shilei Pu, Hai Lin, Yue Zhao, Zhipeng Li, Weitian Zhang

**Affiliations:** ^1^ Department of Otolaryngology-Head and Neck Surgery, Shanghai Sixth People’s Hospital Affiliated to Shanghai Jiao Tong University School of Medicine, Shanghai, China; ^2^ Otolaryngological Institute, Shanghai Jiao Tong University, Shanghai, China; ^3^ Shanghai Key Laboratory of Sleep Disordered Breathing, Shanghai, China; ^4^ School of Environmental Science and Engineering, Shanghai Jiao Tong University, Shanghai, China; ^5^ Department of Otorhinolaryngology Head and Neck Surgery, Shanghai Children’s Hospital, School of Medicine, Shanghai Jiao Tong University, Shanghai, China

**Keywords:** fine particulate matter, organic components, chronic rhinosinusitis, fibroblast, cell migration inducing hyaluronidase 1

## Abstract

**Background:**

Fine particulate matter (PM_2.5_) is harmful to respiratory health and can lead to chronic rhinosinusitis (CRS). But how its components lead to CRS by affecting the function of nasal fibroblasts remains unclear.

**Methods:**

In this study, polar organic components (P-OC) and non-polar organic components (N-OC) isolated from PM_2.5_ were used to intervene human nasal fibroblasts (hNFs), respectively. CCK8 assay and LDH assay were used to detect cell viability, and scratch assay was used to detect cell migration ability. Gene expression changes were detected by RNA-sequencing and molecular biology.

**Results:**

P-OC inhibited the viability and migration of hNFs, while N-OC significantly promoted the migration of hNFs. The expression of CEMIP, MMP1 and IL-1β was upregulated after N-OC treatment. CEMIP gene silencing inhibited hNFs migration and the expression of MMP1 and IL-1β. The effect of PAHs exposure on cells was more obvious than n-alkanes.

**Conclusion:**

N-OC in PM_2.5_, especially PAHs, can aggravate CRS by activating hNFs through CEMIP. This study provides new ideas for exploring the pathogenic mechanism of air pollution on upper respiratory diseases.

## Highlights

1. OCs with different polarity in PM_2.5_ have different effects on hNFs.
2. N-OC promoted hNFs migration and activated fibroblast function.
3. N-OC up-regulates CEMIP and induces MMP1 and IL-1β expression.
4. Among N-OC, PAHs have a stronger effect on hNFs gene expression.

## 1 Introduction

Air pollution, particularly PM_2.5_, is a significant environmental health concern due to its potent pathogenic effects. The respiratory tract is the first to be exposed to PM_2.5_, so it is the most vulnerable to PM_2.5_ damage. Epidemiological studies have shown that exposure to PM_2.5_ substantially raises the incidence of pulmonary fibrosis, asthma, chronic obstructive pulmonary disease, and lung cancer (Atkinson et al., 2014; Soriano et al., 2017). Chronic rhinosinusitis (CRS) is a prevalent airway disease that significantly impacts patients’ quality of life. Studies have shown that PM_2.5_ exposure is associated with the progression of CRS and the worsening of clinical symptoms, although the exact mechanisms remain poorly understood (Mady et al., 2018; Yang et al., 2022).

Inflammation and tissue remodeling are central processes in the onset and progression of CRS. In previous studies, PM_2.5_ exposure has been found to promote inflammation in CRS (Lee and Kim, 2022; Lubner et al., 2024). Zhao R et al. found that compared with normal or CRS model rabbits, the number of fibroblasts in the nasal mucosa tissue of PM_2.5_ exposed rabbits increased, and collagen deposition aggravated (Zhao et al., 2018), which are characteristics of tissue remodeling. Fibroblasts play a critical role in both inflammation and remodeling within CRS, and as such, have attracted increasing attention in recent years (Ball et al., 2016; Shin et al., 2023). Therefore, we suggest that PM_2.5_ may aggravate inflammation and matrix remodeling in CRS by affecting fibroblast function. Numerous studies have highlighted the role of PM_2.5_ in promoting pulmonary fibrosis through fibroblast activation and tissue remodeling (Xu et al., 2019; Nie et al., 2022). However, research on the upper respiratory tract has predominantly focused on the impact of PM_2.5_ on cell viability and inflammatory responses, while the critical process of matrix remodeling has been less frequently explored (Lee et al., 2018; Kim et al., 2020a; Kim et al., 2020b).

PM_2.5_ is a complex mixture of black carbon, gravel, inorganic salts and a variety of organic components (OCs). Previous studies have indicated that PM_2.5_ from different sources have distinct health effects (Kazemiparkouhi et al., 2022; Kobayashi et al., 2023; Zhang et al., 2023), likely attributable to variations in their composition (Bell et al., 2009; Landkocz et al., 2017; Korsiak et al., 2022; Yan et al., 2024). Among them, insoluble components mainly cause physical damage to the epithelium, while fat-soluble OCs may provide stronger chemical toxicity. Especially for fibroblasts that are not in direct contact with PM_2.5_ particles, it may be more meaningful to explore the effect of chemical stimulation of fat-soluble OCs on cell function.

Therefore, we hypothesize that certain OCs of PM_2.5_ cause CRS by affecting the function of human nasal fibroblasts (hNFs). To verify this hypothesis, we collected PM_2.5_ particles from the Shanghai area and identified their organic content. According to the polarity of organic matter, we further divided the OCs in PM_2.5_ into polar organic components (P-OC) and non-polar organic components (N-OC). hNFs cultured *in vitro* were stimulated using P-OC or N-OC, respectively, to compare the effects of different OCs on cells. Cell migration was detected by scratch assay, and related gene expression changes were identified and validated by RNA-seq and molecular biology methods. Our results showed that N-OC in PM_2.5_ promoted hNFs migration and induced matrix metallopeptidase 1 (MMP1) and interleukin-1β (IL-1β) expression by up-regulating cell migration inducing hyaluronidase 1 (CEMIP) expression. It is suggested that the N-OC of PM_2.5_, especially polycyclic aromatic hydrocarbons (PAHs), may aggravate inflammation and tissue remodeling in CRS by activating the function of hNFs.

## 2 Materials and methods

### 2.1 Subjects

hNFs were isolated from nasal septal tissue obtained from 12 patients without chronic airway inflammatory diseases undergoing septal surgery. Each specimen was cultured independently. Due to the limited cell numbers, not all cells were used for all experiments. For details, see the Supplementary Appendix S1. This study was approved by the Ethical Committee of Shanghai Sixth People’s Hospital Affiliated with Shanghai Jiao Tong University School of Medicine, and all subjects provided informed consent.

### 2.2 PM_2.5_ sampling

PM_2.5_ samples were collected on the rooftop of a 20-m-high building (31.201°N, 121.429°E) on Xuhui Campus of Shanghai Jiao Tong University during winter 2023 (December to January). The sampling site is located in central Shanghai, a coastal city with a north temperate monsoon climate. The site itself is open and free of significant local pollution sources. Sampling was performed each day for 23 h (9:00 to 8:00 the next day) onto an 8*10-inch quartz fiber filter (Whatman, United Kingdom) using a sampler (HiVol 3,000, Ecotech) with a flow rate of 1.0 m^3^/min.

Ambient PM_2.5_ concentrations were obtained from the Xuhui campus of Shanghai Normal University, located 4.5 km southwest of the sampling site. Filters from three polluted days (PM_2.5_ > 75 μg/m^3^) were selected for subsequent experiments. Organic carbon was determined using a thermal–optical multiwavelength carbon analyzer (DRI Model 2015), and organic matter (OM) concentration was estimated by multiplying the organic carbon value by a factor of 1.6.

### 2.3 Chemical characterization of PM_2.5_ samples

The methods for extracting organic substances and determining their components are based on the studies by Hu et al. (2019) and Hu et al. (2008). N-OC and P-OC were extracted from the filtered membrane samples using dichloromethane and methanol. Gas chromatography-mass spectrometry (GC-MS) (QP 2020, Shimadzu) equipped with SH-5MS column (30 m length, 0.25 mm diameter, 0.25 μm film thickness, J&W Scientific) was used to analyze all samples under selected ion monitoring (SIM) mode. For details, see the Supplementary Appendix S1.

### 2.4 PM_2.5_ extraction and sample preparation for biological assays

Except for the area used for chemical analysis, all the remaining filter membranes were used for extracting organic components. The filter membranes were cut and then immersed into 3 ml dichloromethane twice by sonication for 30 min in an ice bath. The extracts obtained each time were added together and filtered through a 0.45 μm polytetrafluorethylene syringe filter (CNW Technologies GmbH). The filtrate was dried at 40 °C and redissolved with 1 ml dimethyl sulfoxide (DMSO) to obtain the N-OC solution for the experiment. The filter residue was then immersed again into 3 ml methanol, sonicated, filtered, dried, and dissolved in DMSO as described above, to produce the P-OC solution for the experiment.

### 2.5 Cell culture and exposure

hNFs were extracted from the nasal mucosa of healthy control subjects. Tissues were rinsed in PBS containing antibiotics and antimycotics, minced, and placed on type I collagen-coated dishes. Cells were cultured in DMEM-F12 supplemented with 10% FBS at 37 °C and 5% CO_2_. Cells cultured to passages 3-8 were used for subsequent experiments.

Prior to interventions, the culture medium was replaced with serum- and antibiotic-free DMEM-F12 to avoid experimental interference. For PM_2.5_ exposure, P-OC or N-OC were diluted in DMSO to specified concentrations and applied at 1% (v/v) (based on preliminary experiments) for 6, 18, or 24 h. PAHs mixed standard (16 compounds, 2000 mg/L each) (EPA8100/EPA610/HJ805, Anpel) and n-alkanes mixed standard (C7–C40, 1,000 mg/L each) (HJ894-2017, Anpel) were similarly diluted in DMSO to concentrations reflecting those measured in N-OC solutions, and also applied at 1% (v/v) for 24 h.

### 2.6 Cell viability assay

The Cell Counting Kit-8 (CCK8) was used to detect the cell viability of hNFs, and lactate dehydrogenase (LDH) assay was used to detect cytotoxicity. For details, see the Supplementary Appendix S1.

### 2.7 Cell migration assessment

The migration ability of hNFs was assessed using a scratch assay. Cells were seeded into 12-well plates and cultured to full confluence. A uniform scratch was generated in the center of each well using a 1 mL pipette tip. After washing with PBS to remove debris, hNFs were treated with DMSO, P-OC or N-OC immediately and cultured in serum-free DMEM-F12 medium to minimize the contribution of cell proliferation to wound closure. Images of the same scratch region were captured at 0, 6, 18, and 24 h under a light microscope. The scratch area was measured with ImageJ, and the percentage reduction relative to the 0 h time point was calculated to quantify cell migration.

### 2.8 RNA sequencing

Total RNA was extracted from cells using TRIzol^®^ Reagent following the manufacturer’s instructions. Subsequent RNA purification, reverse transcription, library construction, and sequencing were conducted byMajorbio Bio-pharm Biotechnology Co., Ltd. (Shanghai, China). Gene expression was quantified using the transcripts per million (TPM) method. Differential expression genes (DEGs) between exposure groups (P-OC or N-OC) and the control group (DMSO) were identified with thresholds of |log2FC| ≧ 1 and FDR <0.05. Significantly enriched GO terms among DEGs were identified at a bonferroni corrected p-value <0.05. For details, see the Supplementary Appendix S1. Raw expression data have been deposited in the Gene Expression Omnibus (GEO) database (GEO accession number: GSE284720).

### 2.9 Short interfering RNA transfection

siRNA transfection was carried out using Lipofectamine RNAiMAX. The RNA-lipid complex was prepared by combining 3 μl transfection reagent and 100 nM siRNA in 500 μl total volume, incubated for 20 min at room temperature, and then mixed with 500 μl of hNEs cell suspensionbefore plating in 12-well plates. The final concentration of siRNA was 50 nM. After 24 h, the transfection mixture was replaced with serum-free DMEM-F12 medium, and cells were cultured for another 1–3 days for subsequent experiments.

### 2.10 RNA extraction and reverse transcription quantitative polymerase chain reaction (RT-qPCR)

Total RNA was extracted from cells using TRIzol reagent (Invitrogen). A total of 1 μg RNA was reverse-transcribed to cDNA using a Color Reverse Transcription Kit (A0010CGQ, EZBioscience). Quantitative PCR was performed with the SYBR Green Ⅰ method by using Color SYBR Green qPCR Master Mix (ROX2 plus) (A0012-R2, EZBioscience) with specific primers (Supplementary Table S1). Beta-glucuronidase (GUSB) was used as an internal control for normalization of gene expression. The relative mRNA expression was calculated by using the 2^(−ΔCt)^ method. For details, see the Supplementary Appendix S1.

### 2.11 Western blot (WB)

Cell lysates or supernatant were run on 4%–12% gradient SDS-PAGE gels under reducing conditions. WB was performed using primary antibodies against CEMIP, MMP1, IL-1β, and GAPDH. Densitometric analysis was performed using ImageJ software. The densitometry of CEMIP, MMP1, IL-1β were presented as a ratio over GAPDH. For details, see the Supplementary Appendix S1.

### 2.12 Enzyme linked immunosorbent assay (ELISA)

IL-1β protein levels were measured using a quantitative sandwich ELISA kit according to the manufacturer’s instructions. For details, see the Supplementary Appendix S1.

### 2.13 Statistical analysis

The experimental data were graphed using GraphPad Prism 9.5.0 (GraphPad Software). Normally distributed data were presented as mean ± SEMs, and non-normally distributed data were presented as median (IQR, 25%–75%). Grouped data were analyzed by 2way ANOVA. For column data, Friedman test was used. For X-Y data, simple linear regression was used to analyze data correlation. P < 0.05 was considered statistically significant.

## 3 Results

### 3.1 Composition characteristics of PM_2.5_ during heavy pollution days in Shanghai

To characterize the chemical composition of the collected PM_2.5_, we measured the PM_2.5_ composition in the atmosphere and in solution.

**FIGURE 1:**
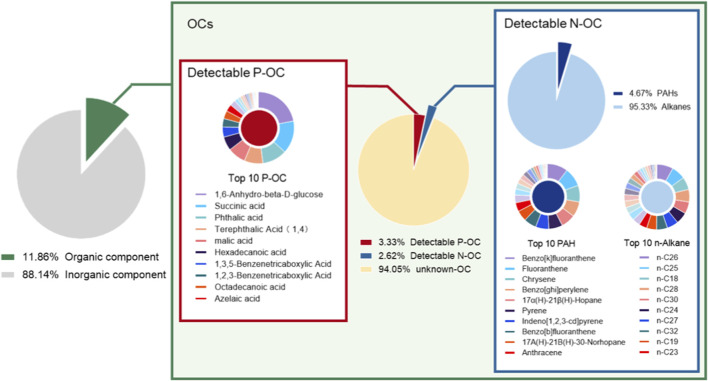
The composition of PM_2.5_ used in the experiment.

During the collection period, the average concentration of PM_2.5_ was 98.55 μg/m^3^, with OM accounting for 11.86% (11.69 μg/m^3^) of the total mass of PM_2.5_ (Figure 1). In the organic extract solution, the total OM concentration was 2.46 mg/ml. Among the identified components (5.95% of total OM), P-OC accounted for 3.33%, and N-OC for 2.62%, while 94.05% remained unidentified (Figure 1). In detected P-OC, the median P-OC concentration was 610.95 ng/ml (IQR: 322.46-3,015.75), with 1, 6-dehydration-β-D-glucose, succinic acid, and phthalic acid being the top three concentrations (Supplementary Table S2). Within the detectable N-OC, PAHs constituted 4.67%, and n-alkanes 95.33% (Figure 1). The median concentrations were 1917.41 ng/ml (IQR: 851.89-3163.04) for n-alkanes (predominantly n-C26, n-C25, and n-C18) and 76.85 ng/ml (IQR: 27.38-190.48) for PAHs (mainly benzo [k]fluoranthene, fluoranthene, and Chrysene) (Supplementary Table S3).

### 3.2 N-OC enhanced hNFs migration

To investigate the effects of P-OC and N-OC, hNFs were treated with varying concentrations and durations of P-OC or N-OC.

**FIGURE 2:**
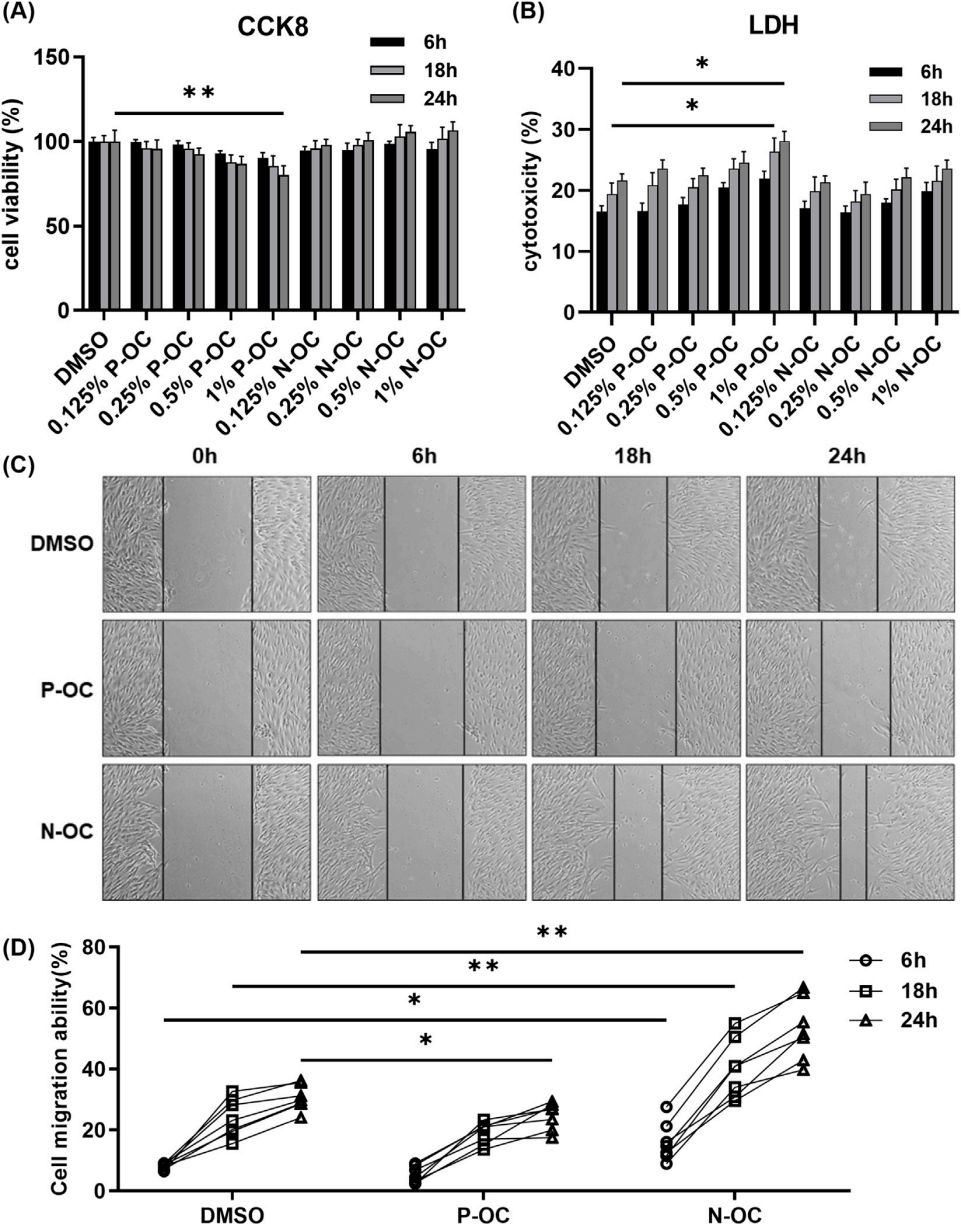
Cell migration capacity increased after stimulation with 1% N-OC concentration. **(A)** hNFs were treated with different concentrations of P-OC or N-OC, and CCK8 was used to detect cell proliferation at different time points. **(B)** The cytotoxicity of different concentrations of P-OC or N-OC was detected by LDH assay after hNFs were intervened for different times. **(C)** After exposure with different conditions, cell morphology and scratch healing were observed under microscope at each time point. **(D)** Statistical analysis of the percentage reduction in the scratch area. *p < 0.05, **p < 0.01.

CCK8 assay revealed that 1% P-OC significantly reduced hNFs viability after 24 h (p = 0.0083), whereas N-OC showed no significant effect (Figure 2A). LDH assay indicated that P-OC induced time- and dose-dependent cytotoxicity, with significant effects at 1% concentration after 18 h (p = 0.0132) and 24 h (p = 0.0244) compared to the DMSO control. In contrast, N-OC exhibited no significant cytotoxicity (Figure 2B). Scratch assay demonstrated that 1% P-OC inhibited wound healing at 24 h (p = 0.0489) compared with DMSO (Figures 2C,D). However, 1% N-OC significantly promoted wound closure at 6 h (p = 0.0319), 18 h (p = 0.0079), and 24 h (p = 0.0013), which is primarily attributable to enhanced cell migration (Figures 2C,D).

### 3.3 N-OC induces transcriptomic alterations in hNFs promoting migration and inflammation

Based on dose-response results, we determined the exposure concentration and time for subsequent experiments. To explore the mechanisms of PM_2.5_ on hNFs, RNA-seq was performed on hNFs treated with 1% P-OC or N-OC for 24 h.

**FIGURE 3:**
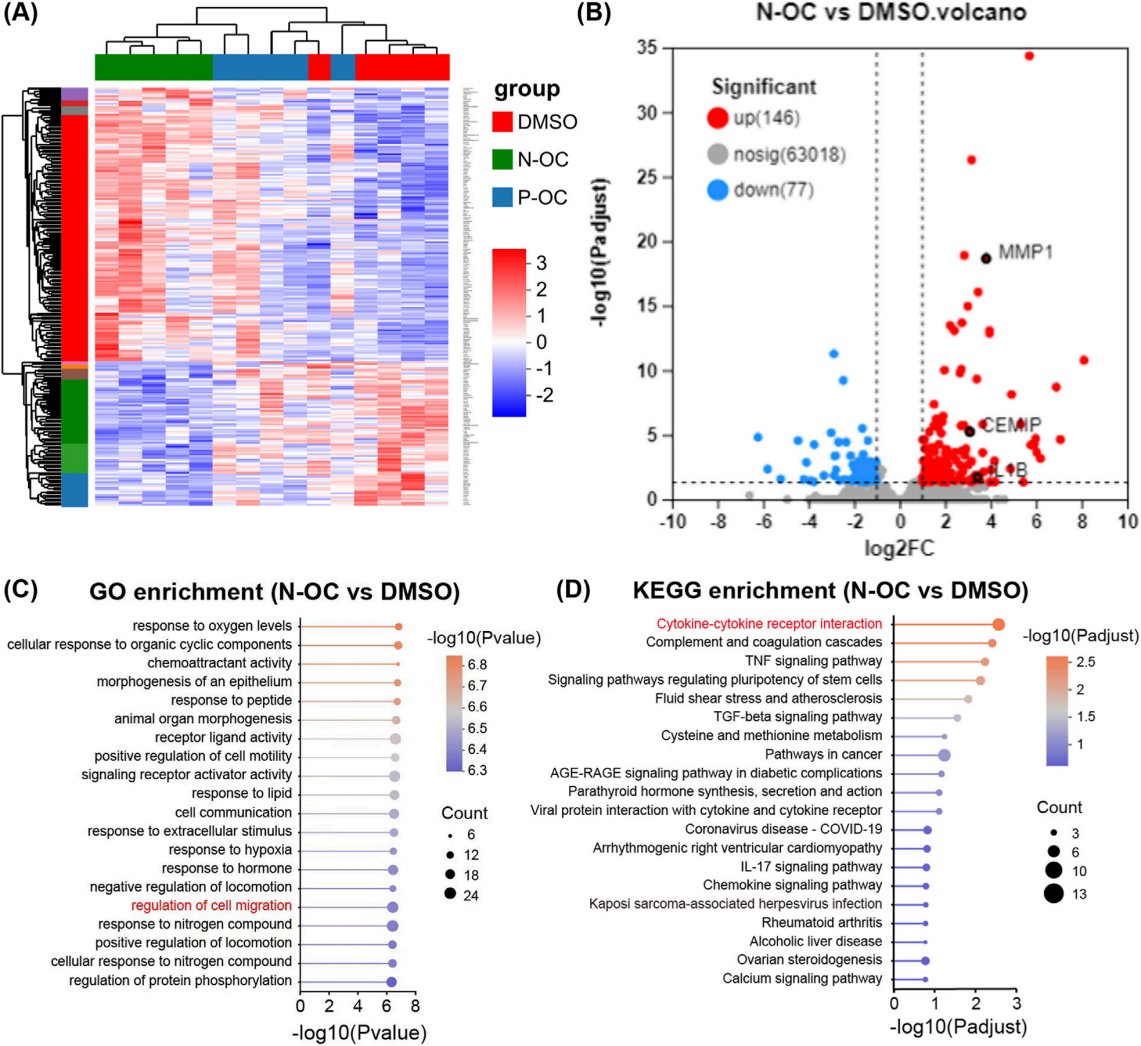
Results of RNA-seq and bioinformatics analysis. **(A)** Heatmap of DEGs. **(B)** Volcano map of DEGs between N-OC and DMSO group. **(C)** GO enrichment analysis of DEGs between N-OC and DMSO group. **(D)** KEGG enrichment analysis of DEGs between N-OC and DMSO group.

The gene expression profile of hNFs treated with N-OC was different from DMSO and P-OC groups (Figure 3A). Consistently, 223 DEGs (146 up-regulated and 77 down-regulated) were identified in N-OC group comparing with DMSO group, much more than DEGs between P-OC and DMSO groups (28 up-regulated and 2 down-regulated) (Supplementary Figure S1A; Supplementary Table S4, 5). Volcano plot further confirmed stronger transcriptomic changes in the N-OC group than in P-OC (Figures 3B; Supplementary Figure S1B), indicating a more pronounced effect of N-OC on hNFs comparing to P-OC. Therefore, in the subsequent analysis, we focused on the functions of the DEGs in the N-OC group.

GO enrichment analysis showed that the DEGs in N-OC group can be enriched to pathways related to cell migration (Figure 3C), consistent with the previous scratch assay results. Among the DEGs enriched in cell migration pathways, we noticed CEMIP and MMP1, two genes encoding enzymes involved in matrix degradation. As shown in Figure 3B, MMP1 and CEMIP were both significantly up-regulated after N-OC stimulation. In addition, KEGG enrichment analysis showed that the differentially expressed genes in the N-OC group could be enriched in the “inflammatory factor” pathway (Figure 3D). Therefore, we also focused on the expression of inflammatory cytokines. We found that the expression of IL1B was also significantly upregulated after N-OC exposure (Figure 3B).

### 3.4 The expression of CEMIP, MMP1, and IL-1β was upregulated in hNFs after 24 h of N-OC stimulation

To validate the results of RNA-seq, hNFs were cultured and treated with 1% DMSO, P-OC or N-OC for 24 h, and the expression of CEMIP, MMP1, and IL-1β was assessed using RT-qPCR, WB and ELISA.

**FIGURE 4:**
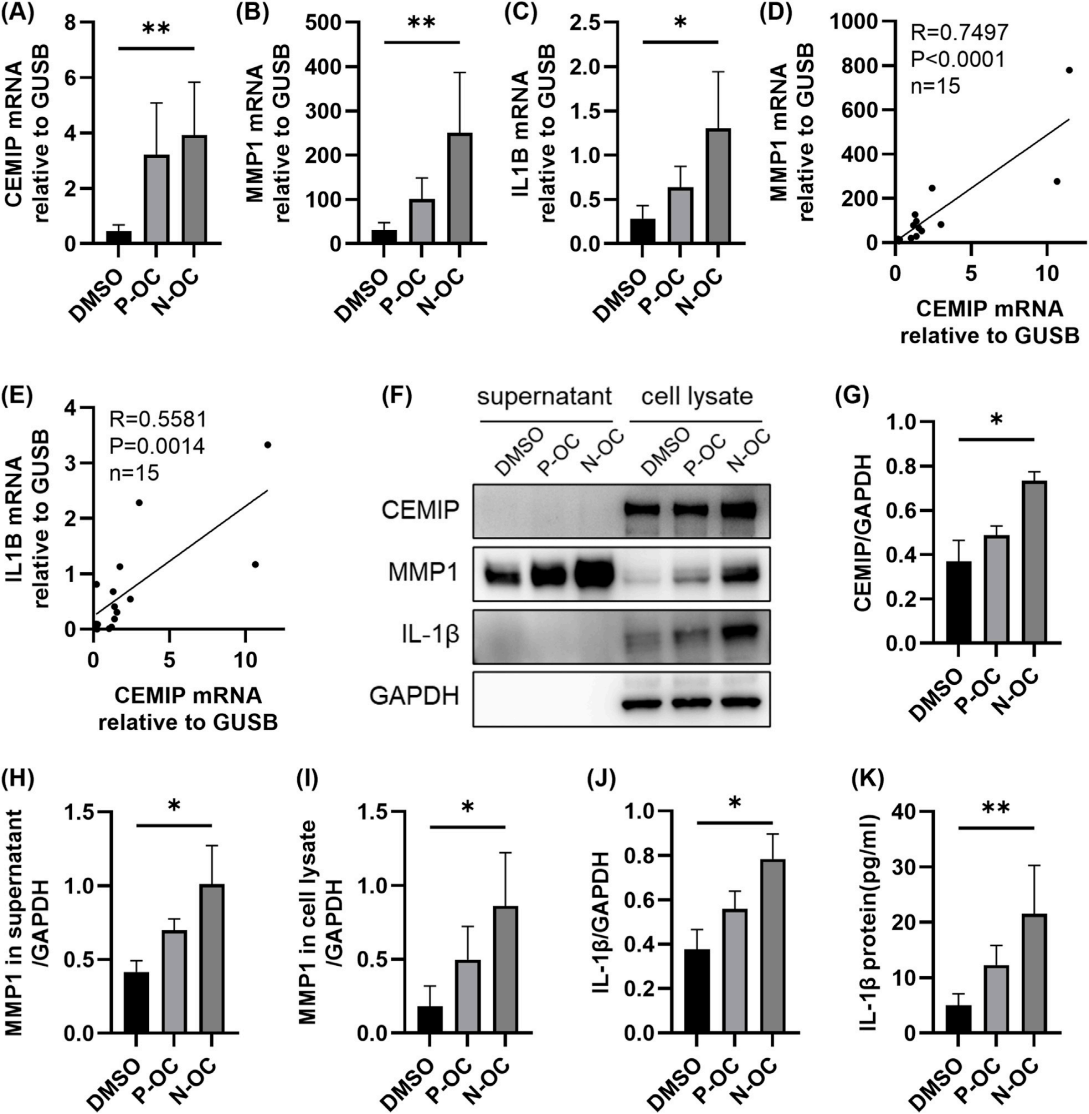
The expressions of CEMIP, MMP1, and IL-1β in hNFs. **(A)** The mRNA expression levels of CEMIP in hNFs by means of RT-qPCR. **(B)** The mRNA expression levels of MMP1 in hNFs by means of RT-qPCR. **(C)** The mRNA expression levels of IL1B in hNFs by means of RT-qPCR. **(D)** MMP1 mRNA expression correlated with CEMIP mRNA expression in hNFs. **(E)** IL1B mRNA expression correlated with CEMIP mRNA expression in hNFs. **(F)** The protein expression levels of CEMIP, MMP1 and IL-1β in hNFs by means of WB. **(G)** Densitometric analysis of CEMIP protein in hNFs by using WB. **(H)** Densitometric analysis of MMP1 protein in supernatant by using WB. **(I)** Densitometric analysis of MMP1 protein in cell lysate by using WB. **(J)** Densitometric analysis of IL-1β protein in hNFs by using WB. **(K)** The expression of IL-1β protein in hNFs was detected by ELISA. *p < 0.05, **p < 0.01.

RT-qPCR analysis showed that exposure to N-OC significantly increased the mRNA expression of CEMIP (p = 0.0047), MMP1 (p = 0.0047), and IL1B (p = 0.0133) compared to the DMSO control (Figures 3A–C). Furthermore, the mRNA levels of MMP1 (Figure 3D, p < 0.0001) and IL1B (Figure 3E, p = 0.0014) were significantly positively correlated with mRNA levels of CEMIP. Consistent with the RT-qPCR results, WB analysis also showed that protein expression of CEMIP was significantly upregulated after N-OC exposure (Figures 4F,G, p = 0.0400). MMP1 protein was detectable in both cell culture supernatants and cell lysates (Figure 4F), and its expression were significantly elevated in both the supernatant (Figure 4H, p = 0.0400) and lysate (Figure 4I, p = 0.0400) after N-OC exposure compared with the DMSO group. Similarly, IL-1β protein expression was significantly increased as measured by WB (Figures 4F,J, p = 0.0400) and ELISA (Figure 4K, p = 0.0045).

It was found that the gene expression of CEMIP, MMP1 and IL-1β was also upregulated after P-OC exposure, but none of them showed statistical difference compared with DMSO or N-OC group (Figure 4).

### 3.5 hNFs migration ability was decreased and the expression of MMP1 and IL-1β was down-regulated after CEMIP knockdown

To investigate whether CEMIP regulates the expression of MMP1 and IL-1β, we silenced CEMIP expression in hNFs using siRNA, and assessed the effects via RT-qPCR, WB and ELISA.

**FIGURE 5:**
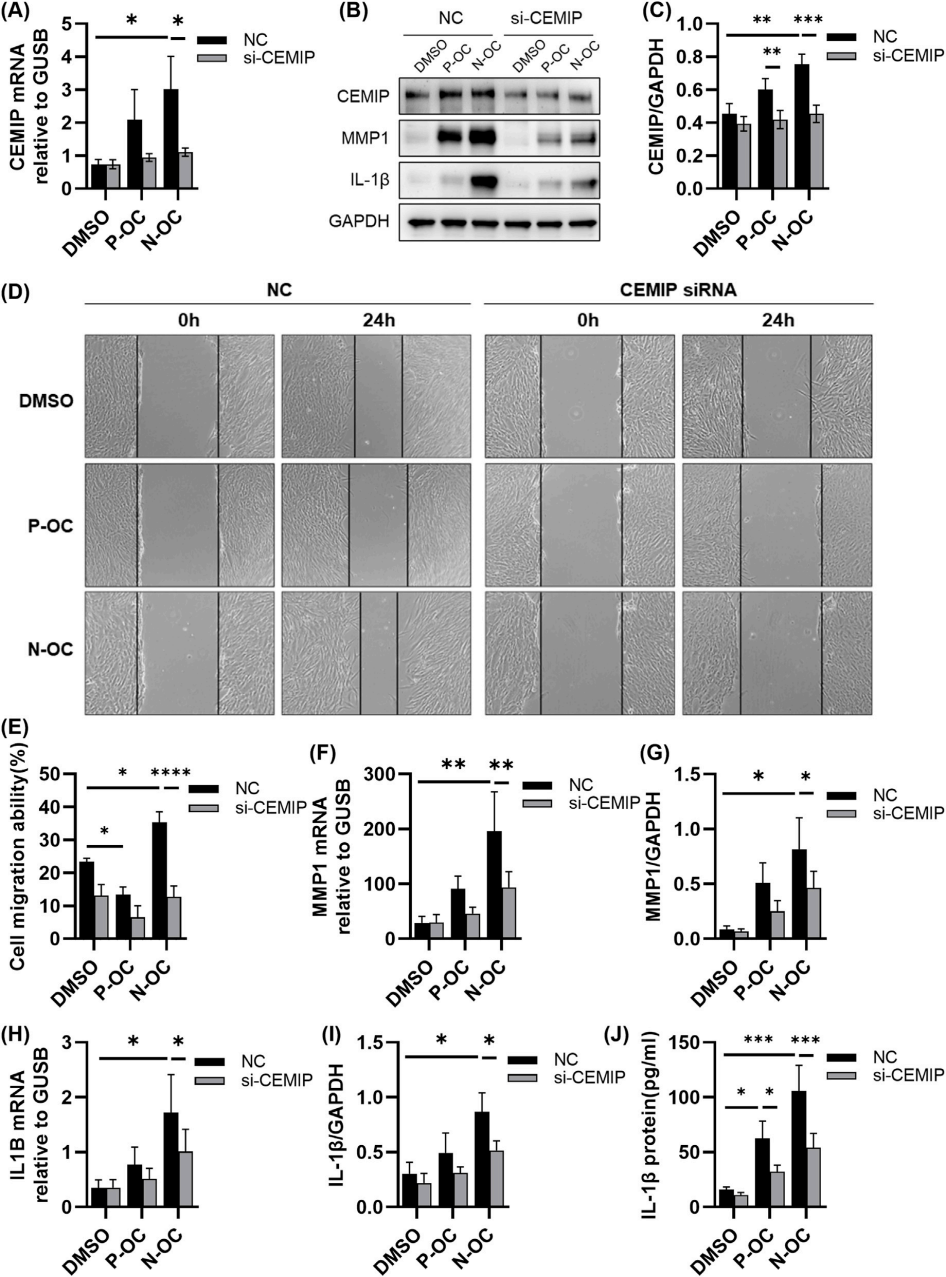
hNFs with or without CEMIP silencing were treated with P-OC or N-OC for 24 h. **(A)** The mRNA expression levels of CEMIP in hNFs by means of RT-qPCR. **(B)** The protein expression levels of CEMIP, MMP1 and IL-1β in hNFs by means of WB. **(C)** Densitometric analysis of CEMIP protein in hNFs by using WB. **(D)** Cell morphology and scratch healing were observed under microscope. **(E)** Statistical analysis of the percentage reduction in the scratch area. **(F)** The mRNA expression levels of MMP1 in hNFs by means of RT-qPCR. **(G)** Densitometric analysis of MMP1 protein in hNFs by using WB. **(H)** The mRNA expression levels of IL1B in hNFs by means of RT-qPCR. **(I)** Densitometric analysis of IL-1β protein in hNFs by using WB. **(J)** The expression of IL-1β protein in hNFs was detected by ELISA. *p < 0.05, **p < 0.01, ***p < 0.001.

RT-qPCR analysis confirmed that the mRNA level of CEMIP could not be upregulated by N-OC stimulation after CEMIP knockdown (Figure 5A, p = 0.0497). WB results showed that the protein levels of CEMIP were inhibited in both P-OC group (p = 0.0046) and N-OC group (p = 0.0001) after CEMIP knockdown, proved that CEMIP gene silencing was successful (Figures 5B,C).

The scratch assay revealed that CEMIP knockdown significantly impaired the migration ability of hNFs exposed to N-OC (Figures 5D,E, p < 0.0001). Moreover, MMP1 mRNA levels in hNFs were significantly reduced in the N-OC exposure group after CEMIP knockdown (Figure 5F, p = 0.0158), which was also confirmed at the protein level by WB results (Figures 5B,G, p = 0.0112). Similarly, IL1B mRNA (Figure 5H, p = 0.0233) and protein levels of IL-1β (Figures 5B,I, p = 0.0220) in the N-OC group were significantly reduced after CEMIP knockdown. The results of ELISA showed that the downregulation of IL-1β protein level caused by CEMIP gene silencing was statistically significant in both the P-OC group (Figure 5J, p = 0.0191) and the N-OC group (Figure 5J, p = 0.0004).

### 3.6 Compared with n-alkanes, the effect of PAHs on CEMIP, MMP1 and IL1B gene expression in hNFs was more pronounced

Given the stronger effect of N-OC compared to P-OC on hNFs, we further investigated the contribution of specific components by treating hNFs with graded concentrations of PAHs or n-alkanes standards for 24 h. Based on the concentrations we measured from N-OC, we diluted the PAHs mixed standards in gradients to concentrations of 5 ng/mL, 25 ng/mL, 100 ng/mL, and 250 ng/mL for each compound. For the n-alkanes mixed standard, due to the high concentration of n-alkanes in N-OC, we set the concentration gradient to 100 ng/mL, 500 ng/mL, 3000 ng/mL, and 5000 ng/mL.

**FIGURE 6:**
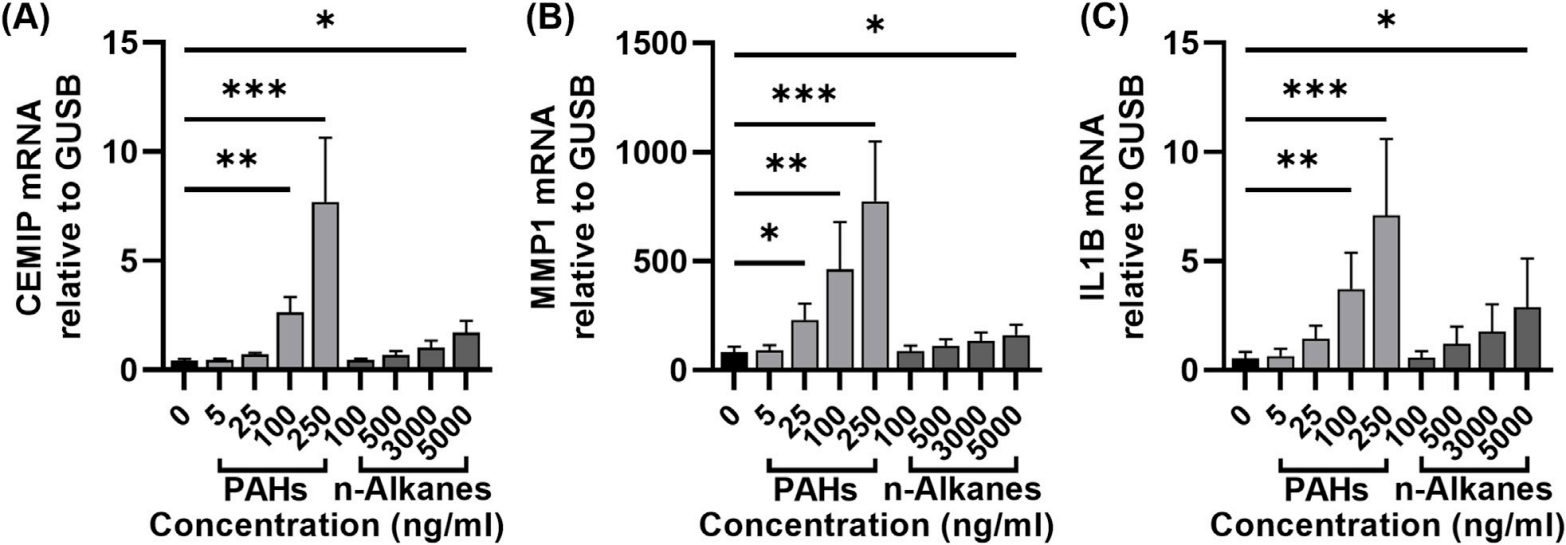
Expression of CEMIP, MMP1 and IL-1β in hNFs after exposure with different concentrations of PAHs or n-alkanes standards. **(A)** The mRNA expression levels of CEMIP in hNFs by means of RT-qPCR. **(B)**The mRNA expression levels of MMP1 in hNFs by means of RT-qPCR. **(C)** The mRNA expression levels of IL1B in hNFs by means of RT-qPCR. *p < 0.05, **p < 0.01, ***p < 0.001.

RT-qPCR analysis revealed that both PAHs and n-alkanes increased the mRNA levels of CEMIP, MMP1, and IL1B in a concentration-dependent manner (Figures 6A–C). PAHs exposure significantly increased MMP1 expression at 25 ng/mL (Figure 6A, p = 0.0361). At higher concentrations (100 ng/mL and 250 ng/mL), PAHs markedly upregulated CEMIP (100 ng/mL p = 0.0024, 250 ng/mL p = 0.0003), MMP1 (100 ng/mL p = 0.0024, 250 ng/mL p = 0.0003) and IL1B (100 ng/mL p = 0.0039, 250 ng/mL p = 0.0003) (Figures 6A–C). However, n-alkanes only induced significant upregulation of all three genes at the highest concentration tested (5000 ng/mL, p = 0.0239, p = 0.0361 and p = 0.0361, respectively). Notably, the magnitude of gene induction by 5,000 ng/mL n-alkanes was lower than that achieved by 100 ng/mL PAHs (Figures 6A–C).

## 4 Discussion

PM_2.5_ is closely linked to the causes and risks of haze, and has consistently been a central focus of international air pollution research. To better understand the health impact of PM_2.5_, recent studies have concentrated on the biological activities of its specific components (Li et al., 2022; Pardo et al., 2022; Moonwiriyakit et al., 2024). In this study, we explored the effects of different OCs of PM_2.5_ on hNFs, and the underlying molecular biological mechanisms. The results showed that P-OC in PM_2.5_ reduced hNFs cell viability and migration ability, whereas N-OC stimulation did not affect cell viability but significantly enhanced cell migration. N-OC exposure, especially the exposure of PAHs, upregulated the expression of CEMIP, MMP1 and IL-1β in hNFs. CEMIP plays a key role in N-OC-induced changes in hNFs function. CEMIP gene silencing can reduce cell migration ability and inhibit the expression of MMP1 and IL-1β.

PM_2.5_ is a significant environmental health concern due to its potent pathogenic effects. It has a wide range of pathological effects by modulating cellular activity and function through various mechanisms, including oxidative stress (as observed in high-level exposed human populations (Hu et al., 2021)), ferroptosis (demonstrated in models of pre-existing pulmonary fibrosis (Yue et al., 2023)), and autophagy (reported in airway epithelial cells (Wu et al., 2020; Liu et al., 2022)). In the case of nasal fibroblasts, previous studies confirmed that PM_2.5_ exposure reduced cell viability in a dose-dependent manner (Lee et al., 2018). Urban PM induces IL-6 and IL-8 expression in hNFs through p38 and NF-κB pathways (Lee et al., 2018), and dose-dependently increases ROS production to activate ERK, thereby elevating HO-1 while reducing SOD2 expression (Kim et al., 2020b). In a 3D-hybrid model, aerosol exposure to PM_2.5_ and Zn resulted in elevated expression of periostin, α-smooth muscle actin, and fibronectin in fibroblast spheroids (Park et al., 2024). In our previous study, the IL-17 signaling pathway in hNFs was activated and the expression of CXC ligand family was up-regulated after PM_2.5_ treatment, suggesting that PM_2.5_ may aggravate inflammatory response through IL-17 signaling pathway in hNFs (Zhu et al., 2024). Many previous studies have confirmed that different components of PM_2.5_ exert distinct pathological effects, but the effects of different components on hNFs are still unclear.

Atmospheric PM_2.5_ consists of various components, such as black carbon, ammonium, nitrate, organic matter, sulfate, soil particles, sea salt, and others (Li et al., 2023). The regional and seasonal variation in PM_2.5_ composition has drawn increasing attention to the differences in the pathological activities of its components. Organic components (OCs), particularly PAHs, in PM_2.5_ demonstrate stronger associations with impaired lung function and higher cytotoxicity than total PM_2.5_ mass (Yan et al., 2024; Yang et al., 2021; Ge et al., 2023), highlighting their critical role in airway dysfunction and attracting increasing research attention (Montgomery et al., 2020; Luo et al., 2021; Moufarrej et al., 2023). In previous studies, OCs of PM_2.5_ have been shown to have pathological activities in airway epithelium, including causing cell death (Bai et al., 2023; Guo et al., 2023; Silva et al., 2023) and DNA damage (Moufarrej et al., 2023; Meng and Zhang, 2007), promoting inflammation (Montgomery et al., 2020; Luo et al., 2021), aggravating epithelial-mesenchymal transition (EMT) (Luo et al., 2021; Chi et al., 2018), and affecting mucociliary function (Montgomery et al., 2020; Deciga-Alcaraz et al., 2023). Notably, even exposed to organic extracts alone produced divergent outcomes across studies, reflecting the high complexity of OC mixtures within PM_2.5_. In our study, we further divided OCs into P-OC and N-OC according to the polarity of the compounds, and compared the pathological activity differences of these two OCs, which is instructive for subsequent studies.

OCs in PM_2.5_ include organic acids, polycyclic aromatic hydrocarbons, n-alkanes and other categories. These organic compounds have different polarities due to their different chemical structures and can therefore be extracted using different organic solvents. Previous studies have shown that OCs of different polarity have different pathological activities on cells, but the conclusions in the current literature are not exactly the same (Reyes et al., 2000; Molinelli et al., 2006; Velali et al., 2016). Reyes DR et al. used n-hexane, dichloromethane, and acetone to extract non-polar, intermediate, and high polar extracts from PM_2.5_ and showed that the non-polar extract extracted with n-hexane was more cytotoxic than the other two extracts (Reyes et al., 2000). Molinelli AR et al. used n-hexane and acetone respectively to extract N-OC or P-OC in PM_2.5_, and found that in addition to being affected by season and collection site, PM_2.5_ cytotoxicity varied with extracts of different polarity (Molinelli et al., 2006). In the study by Velali E et al., there was no significant difference in cytotoxicity between three organic extracts of different polarity, but comet test showed that compared with moderately polar organic fraction and polar organic fraction, non-polar organic fraction caused more severe DNA damage (Velali et al., 2016). In our study, dichloromethane and methanol were used as solvents to successively extract N-OC and P-OC from PM_2.5_ samples. The results showed that compared with DMSO, P-OC exposure promoted cell death and inhibited cell proliferation and migration, whereas N-OC did not affect cell proliferation and death, but instead promoted cell migration. Further RNA-seq also revealed that N-OC induced more significant transcriptomic changes in hNFs. These results suggest that P-OC is more cytotoxic than N-OC, while N-OC has more biological effects.

The variation in cellular responses to PM_2.5_ observed across studies may be attributed to differences in both the types of cells utilized and the specific physicochemical properties of the PM_2.5_ samples employed in the experiments. In terms of the cells used, Reyes DR et al. used human keratinocytes, while Molinelli AR et al. and Velali E et al. both used lung epithelial cell lines, and in our experiments, we used primary human nasal mucosa-derived fibroblasts. It was found that lung epithelial and fibroblast cell lines responded differently to PM_2.5_ after the same concentration of exposure (Liu et al., 2024). Since functional changes of fibroblasts, such as migration, matrix remodeling and inflammatory factor production, rather than death, play a more important role in the pathological process of CRS (8, 9), we focused on the non-lethal pathological activity of N-OC in an attempt to explore the molecular mechanism of its enhanced hNFs migration. In terms of PM_2.5_ samples, our PM_2.5_ samples were collected from developed coastal cities in winter. In the study by Molinelli AR et al., P-OC and N-OC in winter and summer PM_2.5_ from urban sources were used for 48 h of cell exposure to compare the differences in cytotoxicity (Molinelli et al., 2006). While P-OC exhibited greater cytotoxicity in summer samples—consistent with our findings—N-OC showed higher toxicity than P-OC at elevated concentrations in winter, accompanied by a crossover in their dose-response curves (Molinelli et al., 2006). This suggests that even when these two extracts are subdivided into P-OC and N-OC, the differences in the proportions of specific components still seriously affect the cytotoxicity comparison results. Therefore, more studies are still needed to further explore the differences in pathological activities of different OCs in PM_2.5_.

The N-OC in PM_2.5_ mainly includes PAHs and n-alkanes. Previous studies have confirmed the important role of PAHs in the pathogenic of PM_2.5_ (Landkocz et al., 2017; Song et al., 2020). In our study, it was also found that, although PAHs accounted for only a very small fraction of the total mass of N-OC, their alteration of gene expression in hNFs was much stronger than n-alkanes. This suggests that PAHs, even at low concentrations, are key drivers of fibroblast activation in PM_2.5_, likely due to their high biological activity and ability to activate aryl hydrocarbon receptor (AhR) and downstream inflammatory pathways (Polonio et al., 2025). In previous studies, the mechanism by which N-OC or PAHs affect hNFs function remains unclear. By RNA-seq, we found that the gene expression of CEMIP in hNFs was upregulated after N-OC exposure, suggesting that CEMIP may be an important link in the pathological process of CRS induced by N-OC, especially PAHs. Subsequent experiments confirmed that CEMIP is a key gene responsible for N-OC-induced migration in hNFs, likely mediated through its hyaluronidase activity facilitating extracellular matrix degradation (Spataro et al., 2023). Upregulation of MMP1 and IL-1β expression caused by PAHs was also found in our study, which is consistent with the findings of Brinchmann et al. (2018). The expression of MMP1 and IL-1β in hNFs was down-regulated after CEMIP gene silencing, indicating that CEMIP shows more transcription regulator function. These findings are consistent with studies in inflammatory arthritis, where inhibition of CEMIP activity with Ipriflavone reduced TNF-α-induced upregulation of MMP1 and MMP3 (Koike et al., 2022). In another article, silencing CEMIP using shRNA downregulated the RNA levels of IL1B, IL6, IL8, and MMP3 (Deroyer et al., 2022). MMP1 is a collagenase that degrades type I and III collagen, key components of the extracellular matrix (ECM) in nasal mucosa. Its overexpression is associated with tissue remodeling in CRS, leading to basement membrane disruption and stromal fibrosis (Yan et al., 2019; Lygeros et al., 2021). Meanwhile, IL-1β is a potent proinflammatory cytokine that not only amplifies local inflammation but also stimulates fibroblast activation and further ECM remodeling through autocrine and paracrine mechanisms (Shimodaira et al., 2018). Thus, the CEMIP-MMP1-IL-1β axis identified here provides a novel and plausible mechanism for how N-OC, especially PAHs, may aggravate CRS. The synergistic effect of CEMIP-driven cell migration, MMP1-mediated collagen degradation, and IL-1β-sustained inflammation directly contributes to the core pathological features of CRS: dysfunctional tissue remodeling and chronic inflammation.

Although our study focused on the role of CEMIP, it is plausible that other signaling pathways are involved in N-OC-induced fibroblast activation. For instance, NF-κB is a well-known regulator of IL-1β and MMP expression and could be upstream of CEMIP or act in parallel (Shen et al., 2022). Similarly, α-SMA, a marker of myofibroblast differentiation, may be influenced by IL-1β and MMP1 activity (Gabasa et al., 2020). Future studies should explore whether NF-κB, caspase-1 (involved in IL-1β maturation), or TGF-β signaling are activated by N-OC and how they interact with CEMIP.

Our study has several limitations. Firstly, the sample size used was small, but each experiment presented in the article was repeated at least three times with consistent results. Secondly, because the amount of N-OC available is too small to conduct animal experiments, our experiments were only based on *in vitro* studies, and subsequent animal experiments are needed to further verify our conclusions. Finally, we investigated only the effects of high-dose, short-term exposures, whereas real-world exposure often involves low doses over prolonged periods, which may yield different results.

## 5 Conclusion

The present study demonstrated that N-OC, a component of PM_2.5_, may activates the function of hNFs by enhancing their migration and upregulating CEMIP expression. Knockdown of CEMIP attenuated the production of MMP1 and IL-1β, indicating its functional role in N-OC-induced effects. Although PAHs account for only a small fraction of the total mass of PM_2.5_, they play a major role in hNFs activation. These findings improve our understanding of PM_2.5_ exposure on respiratory health, and identify CEMIP as a potential target for future research on air pollution-related airway diseases.

## Data Availability

The data presented in the study are deposited in the Gene Expression Omnibus (GEO) repository, accession number GSE284720.

## Supplementary material

SUPPLEMENTARY FIGURE S1 Results of RNA-seq and bioinformatics analysis. **(A)** The number of differentially expressed genes after comparison between groups. **(B)** Volcano map of DEGs between P-OC and DMSO group.
