# Supplementary material for: Non-polar components in PM2.5 increase matrix remodeling of CRS by up-regulating CEMIP in nasal fibroblasts
Source: Front Genet. 2025 Oct 23;16:1672729. doi: 10.3389/fgene.2025.1672729 (PMC12588579; doi:10.3389/fgene.2025.1672729)
Supplement: Supplementary file 1 [file Supplementaryfile1.docx]

**Materials and methods**

**Subjects**

Surgical specimens used for hNFs cell culture were obtained from patients who underwent nasal septum reshaping but did not have sinusitis, nasal polyps, asthma, allergic rhinitis or other airway inflammatory diseases. During the course of this study, a total of 218 surgical specimens were collected. Among them, 29 cases of nasal septum deviation without nasal inflammatory diseases were included in this study. The remaining specimens with cerebrospinal fluid rhinorrhea, pituitary tumors, nasal polyps, other benign tumors, or those with medical records indicating concurrent rhinitis, sinusitis, or asthma were excluded. Due to the size of the specimens, only 19 samples that met the criteria were used for fibroblast culture. Each sample was cultured independently to avoid contamination from different cell samples. 2 samples were contaminated in the first two generations of culture and were not used in any experiments. A total of 12 specimens were used in this study for cell extraction and culture. 5 samples were used for transcriptome sequencing and all passed quality control. 7 samples were used for other experiments in this study, including scratch assays, PCR, WB, etc. The remaining 5 samples were used for other research by our research group.

**Reagents, Antibodies and Primers.**

Dulbecco’s Modified Eagle Medium/Nutrient Mixture F-12 (DMEM/F-12) and Cell Counting Kit-8 was purchased from MeilunBio (Dalian, China). LDH-Cytotoxicity Assay Kit was purchased from Abnova (Taiwan, China). CEMIP siRNA was from Haixing Biosciences (Suzhou, China), and Lipofectamine RNAiMAX reagent was from Invitrogen (Carlsbad, CA, USA). Color Reverse Transcription Kit (with gDNA Remover) and Color SYBR Green qPCR Mix were purchased from EZBioscience. Primers were purchased from Sangon Biotech (Shanghai, China). GAPDH antibody was purchased from Proteintech (Littleton, CO, USA). KIAA1199 (CEMIP) antibody was purchased from Abcam. MMP1 antibody and IL1B antibody were purchased from Sangon Biotech (Shanghai, China). Human Interleukin-1 beta (IL1B) ELISA Kit was purchased from ABclonal (Wuhan, China). PAHs and n-alkanes standards were purchased from ANPEL Laboratory Technologies (Shanghai, China).

**Chemical Characterization of PM_2.5_ Samples**

Two samples of approximately 17.35 cm^2^ size were cut from these filter membranes and used for chemical analysis. Dichloromethane and methanol were used to extract N-OC and P-OC from the filter membrane samples, respectively.

The extraction of N-OC was referred to the article by Hu H et al^[1]^. For N-OC, prior to extraction, five PAHs (acenaphthene-D_10_, phenanthrene-D10, chrysene-D12, perylene-D12, and naphthalene-D8), one alkane surrogate (C_25_D_50_) were added to the filter membrane samples as internal standards to quantify target compounds. Filter membrane samples were then ultrasonically extracted 3 times with 5 ml dichloromethane for 15 min each time. The resulting extracts were filtered through a PTFE hydrophobic syringe filter (0.45 μm pore size, Millipore) and mixed. The mixed solution was then concentrated to 500 μL using a rotary evaporator (RE52A, Shanghai Yarong Biochemical Instrument), and blow-dried under a gentle nitrogen stream (99.99%, Shanghai Keju Chemical Co., LTD).

The extraction of P-OC was referred to the article by Hu D et al^[2]^. For N-OC, prior to extraction, ketopinic acid, decanoic acid-D19, tetranecanoic acid-D27, phthalate-D4, succinate-D4 were added to the filter membrane samples as internal standards to quantify target compounds. Filter membrane samples were then ultrasonically extracted 3 times with 5 ml methanol for 15 min each time. The resulting extracts were filtered, mixed, concentrated, and blow-dried as previously mentioned. Subsequently, the residue was derivatized with 150 μL of N, O-bis-(trimethylsilyl) trifluoroacetamide (BSTFA)/pyridine (2/1, v/v, Sigma-Aldrich) in an oven at 85°C for 2 h.

GC-MS (QP2020, Shimadzu) equipped with SH-5MS column (30m length, 0.25mm diameter, 0.25 μm film thickness, J&W Scientific) was used to analyze all samples under selected ion monitoring (SIM) mode. For the analysis of P-OC, the GC oven was initially held at 80℃ for 5 min, heated at 3℃ min-1 to 200℃, held for 2 min, then increased to 300℃at 15℃ min-1, and held at 300℃ for 15 min. For N-OC, GC was operated with following oven temperature program: the column temperature started at 60 °C for 1 min, increased to 280 °C at a rate of 14.67 °C/min and maintained for 20 min, increased again to 320 °C at a rate of 8 °C/min and maintained for 2 min. Temperatures of the injector, the ion trap, and the interface were set to 275℃, 200℃, and 300℃, respectively. Known amounts of internal standards were then added before instrumental analysis to calculate the recovery of aforementioned standards.

**Cell viability assay**

Cells were seeded in 96-well plates with 100μl of medium and cultured for 24h. The P-OC or N-OC extracts were sequentially diluted to double, quadruple, and eight-fold volumes using DMSO for subsequent cell experiments. At 24 h, 30 h and 42h after cell inoculation, the cells in different culture Wells were intervened with concentration gradients. 1μl of the corresponding intervention (N-OC or P-OC extract, the three dilutions of gradient dilutions described above) or DMSO was added to each well. At least six complex Wells were set for each concentration at each time. All cells were continued to be cultured in the same environment until 48h for subsequent assays.

Cell viability was detected by Cell Counting Kit-8 (CCK8) (MA0218-2, MeilunBio). Blank Wells, which were not inoculated with cells but only with medium, were set for testing along with the intervention wells. After the above interventions, the medium in each well was aspirated 48h after cell seeding and replaced with a new medium containing 10μl CCK-8 solution, which was incubated for 0.5 to 1 h at 37°C with 5%CO2. Absorbance was measured at 450nm using a microplate reader. The absorbance of each well was subtracted from the average value of the absorbance of blank Wells, and then the ratio was made with the average absorbance of DMSO group. The results were expressed as a percentage to represent the viability of cells in each well.

Cytotoxicity was detected by LDH-Cytotoxicity Assay Kit (KA6677, Abnova). Negative control Wells (added with 10μl ddH2O) and positive control Wells (added with 10 μL provided Tergitol) were also intervened at the same time as each experimental group, and the subsequent experiments were carried out together. At 48h after cell inoculation, 160 µL of Reagent was added to each well and incubated for 10 min at room temperature. Absorbance was measured at a wavelength of 500nm. The absorbance values of all experimental Wells and positive control Wells were subtracted from the mean value of the negative control group, and the results were then compared with the mean value of the positive control group. The results were expressed as a percentage, which was the cytotoxicity of each group.

**RNAseq**

Total RNA was extracted from cell lysates using TRIzol® Reagent according the manufacturer’s instructions. Then RNA quality was determined by 5300 Bioanalyser (Agilent) and quantified using the ND-2000 (NanoDrop Technologies). Only high-quality RNA sample (OD260/280=1.8~2.2, OD260/230≥2.0, RQN≥6.5, 28S:18S≥1.0, >1μg) was used to construct sequencing library.

RNA purification, reverse transcription, library construction, and sequencing were performed at Shanghai Majorbio Bio-pharm Biotechnology Co., Ltd. (Shanghai, China) according to the manufacturer’s instructions. The RNA-seq transcriptome librariy was prepared following Illumina® Stranded mRNA Prep, Ligation using 1μg of total RNA. Shortly, messenger RNA was isolated according to polyA selection method by oligo(dT) beads and then fragmented by fragmentation buffer firstly. Secondly double-stranded cDNA was synthesized using a SuperScript double-stranded cDNA synthesis kit (Invitrogen, CA) with random hexamer primers. Then the synthesized cDNA was subjected to end-repair, phosphorylation and adapter addition according to library construction protocol. Libraries were size selected for cDNA target fragments of 300 bp on 2% Low Range Ultra Agarose followed by PCR amplified using Phusion DNA polymerase (NEB) for 15 PCR cycles. After quantified by Qubit 4.0, the sequencing library was performed on NovaSeq X Plus platform(PE150) using NovaSeq Reagent Kit.

The raw paired end reads were trimmed and quality controlled by fastp^[3]^ with default parameters. Then clean reads were separately aligned to reference genome with orientation mode using HISAT2^[4]^ software. The mapped reads of each sample were assembled by StringTie^[5]^ in a reference-based approach.

To identify DEGs (differential expression genes) between two different samples, the expression level of each transcript was calculated according to the transcripts per million reads (TPM) method. RSEM^[6]^ was used to quantify gene abundances. Essentially, differential expression analysis was performed using the DESeq2^[7]^. DEGs with |log2FC|≧1 and FDR＜0.05 were considered to be significantly different expressed genes. In addition, functional enrichment analysis (including GO and KEGG) was performed to determine which DEGs were significantly enriched in GO terms and metabolic pathways compared to the whole transcriptome background (Bonferroni-corrected P-value＜0.05). Goatools and Python scipy software were used for GO functional enrichment and KEGG pathway analysis, respectively.

**RNA extraction and reverse transcription quantitative polymerase chain reaction (RT-qPCR)**

Total RNA was extracted from cells cultured in vitro using TRIzol reagent (Invitrogen). A total of 1μg RNA was reverse-transcribed to cDNA using a Color Reverse Transcription Kit (A0010CGQ, EZBioscience). Quantitative PCR was performed with the SYBR Green Ⅰ method by using Color SYBR Green qPCR Master Mix (ROX2 plus) (A0012-R2, EZBioscience) with specific primers (Table S1). Amplification was as follows: 95 ℃ for 5 min, followed by 40 cycles of 95 ℃ for 10 sec, specific annealing temperature for 10 sec, and 60 ℃ for 30 sec. After PCR, a melting curve was constructed by increasing the temperature from 60 to 95 ℃ with a temperature transition rate of 0.11℃/s. Beta-glucuronidase (GUSB) was used as an internal control for normalization of gene expression. An identical Ct was applied for each gene of interest. Relative mRNA expression was calculated by using the 2(-ΔCt) method.

**Western blot (WB)**

Cells were lysed in buffer containing protease and phosphatase inhibitors (Thermo Fisher). Proteins (30 μg per lane) were separated on 4–12% SDS-PAGE gels (EpiZyme), transferred to nitrocellulose membranes (Pall) using the eBlot™ L1 system (GenScript), and blocked with 5% non-fat milk for 1 h at room temperature. Membranes were incubated overnight at 4 °C with shaking in primary antibodies against: CEMIP (1:1000; ab308288, Abcam), MMP1 (1:2000; D220093, Sangon Biotech), IL-1β (1:500; D120820, Sangon Biotech), and GAPDH (1:10,000; Proteintech). After incubation with HRP-conjugated secondary antibodies for 1 h at room temperature, signals were visualized by chemiluminescence and analyzed densitometrically using ImageJ.

**ELISA**

ELISA was performed using Human IL-1 beta ELISA Kit (RK00001, ABclonal) according to the manufacturer's instructions. The antibody precoated ELISA plates were washed 3 times using wash buffer and the liquid was patted dry each time. Different concentrations of standards or samples to be tested were added to the Wells and incubated for 2h at 37 ° C. After removing the liquid and repeating the washing step, the Biotin Conjugate Antibody working solution was added and incubated at 37 ° C for 1h. After removing the liquid and repeating the washing step, Streptavidin-HRP working solution was added and incubated at 37 ° C for 1h. After removing the liquid and repeating the washing step, TMB Substrate was added and incubated at 37 ° C in the dark for 15 to 20min. The reaction was terminated by addition of Stop Solution, and the absorbance (OD) at 450 nm was immediately measured with a microplate reader and corrected using absorbance at 630nm.

**References**

1. Hu H, Tian M, Zhang L, Yang F, Peng C, Chen Y, Shi G, Yao X, Jiang C, Wang J. Sources and gas-particle partitioning of atmospheric parent, oxygenated , and nitrated polycyclic aromatic hydrocarbons in a humid city in southwest china. **Atmos Environ (1994)** **2019**, 206: 1-10.

2. Di Hu, Bian Q, Li TWY, Lau AKH, Yu JZ. Contributions of isoprene, monoterpenes, β-caryophyllene, and toluene to secondary organic aerosols in hong kong during the summer of 2006. **J Geophys Res Atmos** **2008**, 113: 14.

3. Chen S, Zhou Y, Chen Y, Gu J. Fastp: an ultra-fast all-in-one FASTQ preprocessor. **Bioinformatics** **2018**, 34(17): 884-890.

4. Kim D, Langmead B, Salzberg SL. HISAT: a fast spliced aligner with low memory requirements. **Nat Methods** **2015**, 12(4): 121-357.

5. Pertea M, Pertea GM, Antonescu CM, Chang T, Mendell JT, Salzberg SL. StringTie enables improved reconstruction of a transcriptome from RNA-seq reads. **Nat Biotechnol** **2015**, 33(3): 290.

6. Li B, Dewey CN. RSEM: accurate transcript quantification from RNA -seq data with or without a reference genome. **BMC Bioinformatics** **2011**, 12: 16.

7. Love MI, Huber W, Anders S. Moderated estimation of fold change and dispersion for RNA-seq data with DESeq2. **Genome Biol** **2014**, 15(12): 38.
