# Supplementary figures and images for: Non-polar components in PM2.5 increase matrix remodeling of CRS by up-regulating CEMIP in nasal fibroblasts
Source: Front Genet. 2025 Oct 23;16:1672729. doi: 10.3389/fgene.2025.1672729 (PMC12588579; doi:10.3389/fgene.2025.1672729)

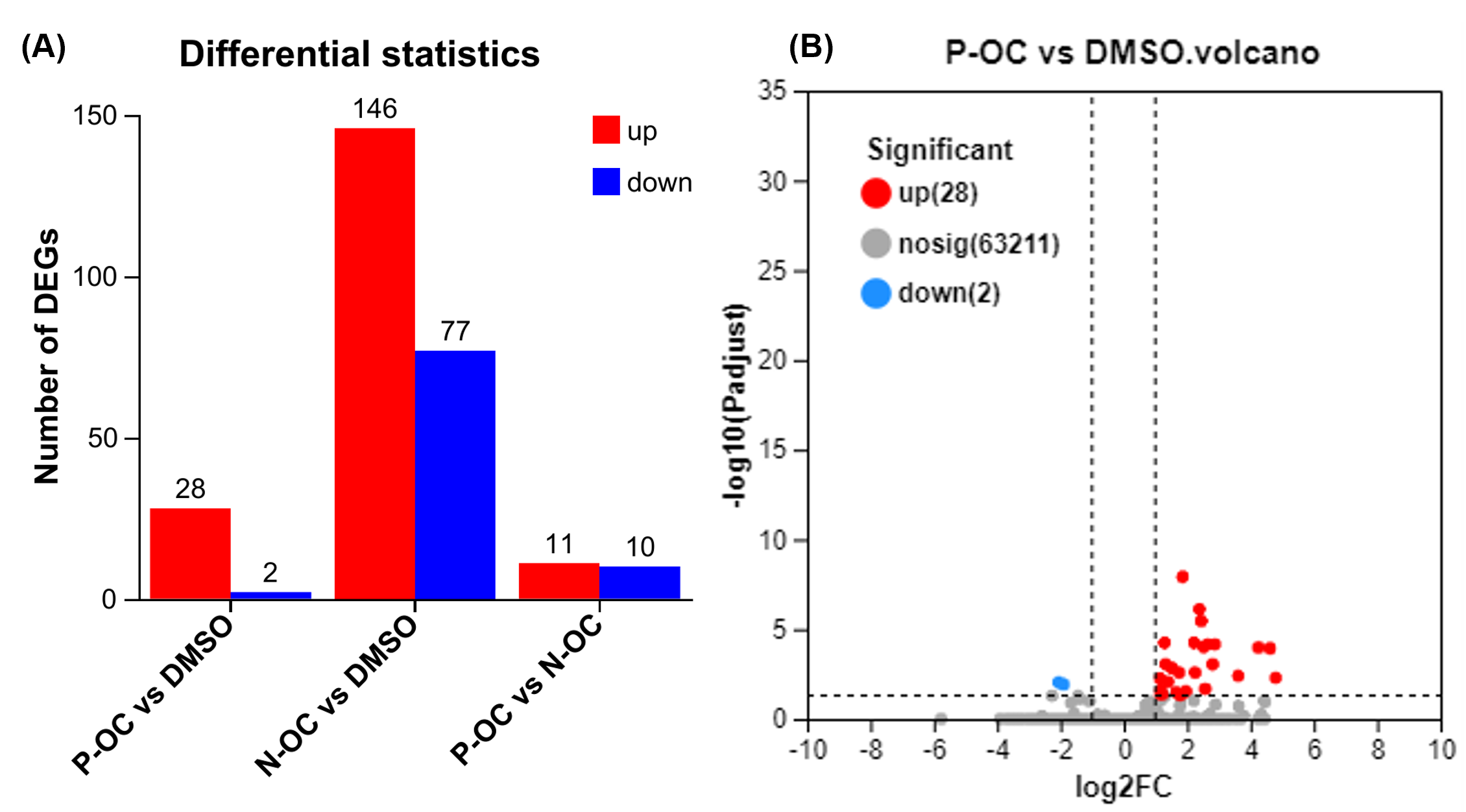

Supplement: Supplementary file 2 [file Image1.tif]
